# Phytochemicals Possess Selective Chemopreventive Mechanisms That Safeguard Human Cells from Oxidative Toxicity

**DOI:** 10.3390/biom16020191

**Published:** 2026-01-27

**Authors:** Annamaria Di Giacomo, Gian Luigi Russo, Stefania Moccia, Carmela Spagnuolo, Maria Russo

**Affiliations:** National Research Council, Institute of Food Sciences, Via Roma 64, 83100 Avellino, Italy; annamaria.digiacomo@isa.cnr.it (A.D.G.); gianluigi.russo@cnr.it (G.L.R.); stefania.moccia@isa.cnr.it (S.M.); carmela.spagnuolo@isa.cnr.it (C.S.)

**Keywords:** phytochemicals, Nrf2, oxidative stress, NQO-1, HO-1

## Abstract

Oxidative stress from environmental pollutants is linked to chronic degenerative diseases. Research indicates that specific phytochemicals in our diets can reduce and mitigate the harmful effects of pro-oxidant insults on health. However, limited randomized clinical trials show the protective effects of these compounds. This lack of in vivo evidence is partly due to the low bioavailability of these compounds, which can obscure their actual benefits. The present work investigates whether selected dietary phytochemicals are equally effective in activating cellular defense against oxidative stress at low doses. In a previous study, we found that Curcumin (Curc) at a concentration of 1 μM protected human myeloid cells from cytotoxicity induced by pro-oxidant species by activating the expression of Nrf2/ARE-dependent transcripts, including NADPH: quinone oxidoreductase-1 (NQO-1) and heme oxygenase-1 (HO-1). Now, we aim to extend our observation to other natural activators of the Nrf2 pathway, such as Sulforaphane (SFN) and three structurally related molecules belonging to the flavonoid family: Quercetin (Q), Catechin (C), and Fisetin (F). These compounds were applied at low concentrations (1 μM) to assess their antioxidant activity against H_2_O_2_-induced oxidative stress, their effects on cellular viability, and the capacity to drive the expression of NQO-1/HO-1 in various cellular models. Our findings indicate that low-dose phytochemicals differ in their cytoprotective efficacy, which depends on both dosage and intracellular uptake or metabolism. We propose that only specific natural antioxidants can protect cells from oxidative stress, underscoring the need to clarify the mechanisms behind this selectivity to better design nutraceuticals and functional foods.

## 1. Introduction

The growing elderly population in Western countries and the simultaneous increase in polluted and unhealthy areas due to industrialization and climate change are key factors in the rise in chronic degenerative diseases [1,2]. Among the lifestyle changes that can help mitigate this phenomenon, nutrition certainly plays a key role [3,4].

A recent paper by Menichetti et al. describes the chemical complexity of food and the numerous metabolites it contains, as well as their implications for therapeutics [5]. Phytochemicals are a class of thousands of biomolecules present in edible plant tissues, belonging to the so-called “nutrition dark matter” (NDM) library, a database comprising thousands of distinct food chemicals, along with their specific structural identifiers, physicochemical properties, and food annotations. Many of these molecules have well-documented health implications, which are evolutionarily derived from their cytoprotective function in plant tissue, as evidenced by human cellular in vitro studies, animal models, or ex vivo studies [6]. Polyphenols, for example, which are absorbed from the diet to varying degrees, can bind to specific human proteins [7], modulate gut microbiota composition [8], and regulate a wide range of cellular and metabolic processes. In other words, with respect to mechanisms of action, the NDM molecules may be closer to drugs than to the molecules linked to energy sources (carbohydrates, proteins, lipids) conventionally studied by nutritional biochemists [6]. However, when randomized case–control clinical trials are conducted to test healthy properties, such as chemopreventive, anti-carcinogenic, antioxidant, and anti-inflammatory effects, very few positive results are obtained. In fact, the main limitation lies in our incomplete understanding of the final fate of phytochemicals after ingestion and their true health effects in different tissues following complex intracellular biotransformation. In addition, to further complicate the field, a major confounding factor in studies using cellular models is that the concentrations of single molecules added to the culture medium often exceed their actual intracellular levels by 10–100-fold, which does not accurately reflect the plasma concentrations of phytochemicals typically achieved through dietary intake. For example, previous studies using resveratrol, a phytoalexin found in peanut seeds, berries, and grapes, employed a concentration of 50–100 μM. In contrast, the actual dose of the free molecule is almost undetectable in human plasma after ingestion with food, and it only reaches a nanomolar concentration after ingestion of 1 g as pure compound [9,10].

In recent years, the concept of hormesis has been integrated to comprise antioxidant, anti-inflammatory, and cellular repair responses at all levels of biological organization (e.g., cell, organ, and organism) within the framework of biphasic dose responses [11]. The rationale behind the hormetic concept is that low levels of biological, chemical, or physical stresses upregulate adaptive responses that not only precondition, repair, and restore normal functions to damaged cells/tissues/organs but also can counterbalance, reducing chronic continuing damage, and thereby enhancing health through a “beneficial” stress. Higher doses of such stress often become counterproductive and eventually deleterious [12]. These cellular responses also correlate with the quantitative limits of biological plasticity in all cells and organisms during the aging process. A theory on the limits of lifespan asserts that aging may diminish hormesis, affecting the onset and severity of debilitating conditions/diseases, especially in the elderly population [11].

Starting from these concepts, the present manuscript aims to investigate the potential cytoprotective effects of selected phytochemicals from edible plants using human cellular models derived from myeloid (differentiated HL-60 and K-562) or intestinal (differentiated HT-29) cells [13,14]. The models were selected for their proven in vitro differentiation capacity and suitability for reproducible analysis of phytochemical mechanisms. The concentration used was the lowest dose to elicit biological activity while remaining close to a nutraceutically relevant level (1 μM) [15,16]. In other words, this is the lower dose, closer to the plasma concentrations of phytochemicals assumed in the diet, capable of producing a measurable biological effect (antioxidant and/or cytoprotective) in our models.

In previous works, we demonstrated that 1 μM Curcumin (Curc) protected myeloid cells from oxidative stress [17]. Here, Quercetin (Q), a well-known flavonoid antioxidant, was used, along with two other flavonoids structurally similar to Q, namely Fisetin (F) [18] and (+) Catechin (C). In parallel, Sulforaphane (SFN) was used as a positive control to assess the involvement of the Nrf2 signaling pathway. Our work embraces previous observations suggesting that, paradoxically yet realistically, dietary phytochemicals may act as bland electrophilic molecules within cells [19,20]. Here, their oxidized metabolites (e.g., oxidized quinones), though non-toxic at low concentrations, can induce an adaptive cellular response to counteract external stressors by activating the Nrf2/ARE-dependent antioxidant pathway [19,21,22].

The Nrf2 system, which involves the translocation of a transcription factor into the nucleus, is not the sole mechanism safeguarding cellular homeostasis in eukaryotic cells during episodes of redox imbalance, particularly at the cell membrane level. One such mechanism is the multienzyme complex known as the Plasma Membrane Redox System (PMRS) [23]. PMRS participates in transferring reducing equivalents from intracellular donors to extracellular acceptors, primarily oxidized ascorbate. By this process, the PMRS assists cells in responding to alterations in redox potential, thus regulating various physiological functions, including cell metabolism, ion channel activity, growth, and apoptosis. Previous research has demonstrated that certain polyphenols can donate reducing equivalents to PMRS, suggesting that their role in PMRS activity warrants further investigation within our screening framework [23].

The results of the present study may ultimately be applied to identify bioactive compounds or food matrices enriched with natural molecules that act on known targets and, if validated in randomized clinical trials, possess realistic therapeutic potential for the prevention of chronic and degenerative diseases.

## 2. Materials and Methods

### 2.1. Cell Culture and Differentiation

HL-60 and K-562 cell lines were acquired from the European Collection of Cell Cultures (ECACC) and distributed in Italy by Merck/Sigma-Aldrich (Milan, Italy) [24,25]. They were cultured in RPMI medium supplemented with 10% FBS, 1% L-glutamine, and 1% penicillin/streptomycin (Euroclone, Milan, Italy) at 37 °C in a humidified atmosphere containing 5% CO_2_. HL-60 cells were induced to differentiate into macrophages after 72 h of supplementation with 100 nM 1α, 25-dihydroxyvitamin D3 (VD) analog EB1082 (Merck/Sigma-Aldrich) [26,27]. K-562 cells were differentiated into erythroblasts by adding 50 μM of resveratrol in the cell culture medium (24–72 h) [28]. HT-29 cells, purchased by ATCC (American Tissue and Cell Culture) bank distributed in Italy by LGC Standards (Milan, Italy) [29], were grown in DMEM high glucose medium supplemented with 10% fetal bovine serum FBS, 1% L-glutamine, and 1% penicillin/streptomycin (Euroclone, Pero, MI, Italy) at 37 °C in a humidified atmosphere containing 5% CO_2_. HT-29 cells, which are induced to differentiate into enterocytic cells by adding 4 mM sodium butyrate for 72 h, exhibit characteristics of mature intestinal cells and form a tight monolayer with an apical brush border, similarly to enterocytes [30].

### 2.2. Study Design and Viability Assays

Curcumin, Sulforaphane, Quercetin, Fisetin, and (+)-Catechin were all purchased from Merck/Sigma-Aldrich. Differentiated cells were preincubated 2 h or overnight with the indicated dose of phytochemicals (1–10 μM) dissolved in Dimethyl Sulfoxide (DMSO Merck/Sigma-Aldrich) or the relative/not toxic amount of DMSO dissolved in medium (0.1–0.2% *v/v* Control, Ctrl). Subsequently, the medium was changed, and H_2_O_2_ (Merck/Sigma-Aldrich) or t-butyl hydroperoxide (0.5 mM) (Merck/Sigma-Aldrich) was added for an additional 30 min to measure intracellular peroxide levels or for 24 h to assess cytotoxicity. Cellular viability was assessed using CyQuant dye (Invitrogen, Life Technologies, Milan, Italy) or Crystal Violet (Merck/Sigma-Aldrich) staining, as described [31]. After differentiation, cellular morphology was documented in HL-60 and K-562 cells by staining with 4-Nitro Blue Tetrazolium chloride (NBT) (Roche/Merck) or Neutral Red solution (Merck/Sigma-Aldrich), respectively, as previously described [17]. Microphotographs were captured using a fluorescence inverted microscope (Axiovert Zeiss, Milan, Italy) at 400× magnification, either with phase contrast or a FITC filter, at the end of the staining process. For HT-29 cells, differentiation towards mature intestinal cells and the formation of a tight monolayer with an apical brush border were assessed by measuring the enzymatic activity of alkaline phosphatase and by microscopical observation (Axiovert Zeiss; 200× magnification) [32].

### 2.3. Intracellular ROS Measurements

Differentiated cells at a concentration of 2 × 10^4^/mL were pre-incubated with different natural molecules for 2 h or overnight. Subsequently, the cells were washed and incubated for 30 min in the presence of the chloromethyl derivative of dichlorofluorescein diacetate (10 µM CM-DCFDA; Invitrogen, Life Technologies). This probe was used to measure intracellular peroxides after the addition of H_2_O_2_ as an external stressor, starting at 5 min and continuing for up to 30 min. The incubation was carried out in PBS or wash buffer at 37 °C in a humidified 5% CO_2_ atmosphere. Fluorescence was measured using a Synergy HT multiwell reader (BioTek, Monza, Italy) with excitation at 485 nm and emission at 530 nm for CM-DCFDA. The results were expressed as DCF fluorescence (%) relative to the fluorescence of untreated cells (Ctrl).

### 2.4. Immunoblotting

The cells were disrupted using a lysis buffer that contained protease and phosphatase inhibitors, as reported [31]. After measuring the protein concentration using the Lowry method [33], protein lysates (20 µg) were mixed with 4× Laemmli loading buffer (Bio-Rad, Milan, Italy). The mixture was heated at 95 °C for 5 min and loaded onto a 10% SDS-PAGE gel at pH 8, as previously described [31]. The immunoblots were conducted using Polyvinylidene Fluoride (PVDF) membranes (Bio-Rad). They were incubated for approximately 16 h with primary antibodies against NQO-1 (Cell Signaling Technologies; distributed by Euroclone, Milan, Italy, catalog number #62262), HO-1 (Cell Signaling Technologies, catalog number #3187), and α-tubulin (Merck/ Sigma-Aldrich, catalog number #T9026). The primary antibodies were diluted 1:1000 in Tween 20-Tris Buffer Saline (T-TBS) containing 3% Bovine Serum Albumin (BSA, Merck/Sigma-Aldrich). The membranes were washed in T-TBS and incubated for 2 h with a horseradish peroxidase-linked secondary antibody raised against mouse or rabbit. The secondary antibody was diluted 1:20,000 in T-TBS. The immunoblots were developed using the ECL Prime Western Blotting Detection System kit (Cytiva, distributed by Euroclone). The band intensities were quantified and expressed as optical density using a Chemidoc apparatus (Bio-Rad) and Image Lab software (Bio-Rad version 6.1).

### 2.5. Quantitative RT-PCR

HL-60 cells and K-562 cells (3 × 10^6^) were induced to differentiate for 72 h with EB 1089 or resveratrol (50 μM) for 24 h, respectively. The medium was changed, and the cells were treated with the selected compounds at different concentrations (1–10 μM) and harvested at various times (120 min–24 h) post-stimulation. Total RNA was extracted from the cells using the Monarch Total RNA Miniprep kit (New England Biolabs, distributed by Euroclone), reverse-transcribed (1 μg, LunaScript RT Supermix 5X, New England Biolabs), and analyzed by quantitative PCR (qPCR) using gene-specific primers (Supplementary Table S1) and Fluocycle IIᵀᴹ SYBR GREEN Master Mix (Euroclone) with an AriaMx instrument (Agilent Technologies, Milan, Italy). The relative levels of gene expression for both target and reference genes were calculated by the 2^−ΔΔCt^ method based on Ct values. The data (*n* = 2–3 independent experiments performed in duplicate/triplicate) were presented as the mean ± standard deviation (SD) of 2^−ΔCt^ values. All mRNA levels were normalized to endogenous GAPDH expression [17].

### 2.6. Alkaline Phosphatase Enzymatic Activity

Alkaline phosphatase (ALP) was measured in total cell extracts using p-nitrophenyl phosphate (p-NPP; Merk/Sigma-Aldrich) as a substrate. Briefly, HT-29 cell extract (5 μg) was incubated at 37 °C for 10 min in a reaction buffer containing 10 mM p-NPP and 5 mM MgCl_2_ in 100 mM Tris/HCl, pH 9.5. The reaction was terminated by adding 1 N NaOH. The extent of hydrolysis was measured spectrophotometrically at 405 nm, and the results were expressed as O.D. (optical density) values/min/μg protein [34].

### 2.7. Plasma Membrane Redox System (PMRS) Enzymatic Assay

To measure PMRS activity, differentiated HT-29 cells (1 × 10^6^/well) were washed with PBS and incubated at 37 °C for 30 min in HBSS (Euroclone) containing the indicated polyphenols dissolved in DMSO. Cells were washed with PBS and treated with a mixture containing PBS, 5 mM glucose, and 1 mM K_3_Fe (CN)_6_ at 37 °C for 30 min. After centrifugation, the supernatants were collected for the PMRS assay as previously reported [23]. Absorbance was measured at 540 nm, and results were expressed as μM ferrocyanide/10^6^ cells/min.

### 2.8. Statistical Analysis

Students’ *t*-test (applied for two groups of data), or One-way ANOVA (applied for multiple data samples), followed by Tukey’s multiple comparisons test, was performed using GraphPad Prism version 9.5.1 for macOS (GraphPad Software, San Diego, CA, USA). Statistical significance was accepted at a *p*-value of less than 0.05. The results are expressed as the mean ± standard deviation (SD) based on values obtained from independent experiments (*n* = 2–3) performed in duplicate, triplicate, or quadruplicate. Specifically, when two biological replicates were used, experiments were performed in triplicate or quadruplicate technical replicates to ensure adequate statistical power for experiments that are not feasible to replicate. The limitation of this analysis is that conclusions from experiments with *n* = 2 biological replicates should be interpreted conservatively. Where applicable, we emphasized consistency across independent experiments rather than relying on formal statistical analysis of significance. Specific values were indicated in figure legends: * *p* < 0.05, ** *p* < 0.01, *** *p* < 0.001; **** *p* < 0.0001.

## 3. Results

### 3.1. Chemical Structure of Dietary Phytochemicals and Experimental Models Used in the Screening

**Figure 1:**
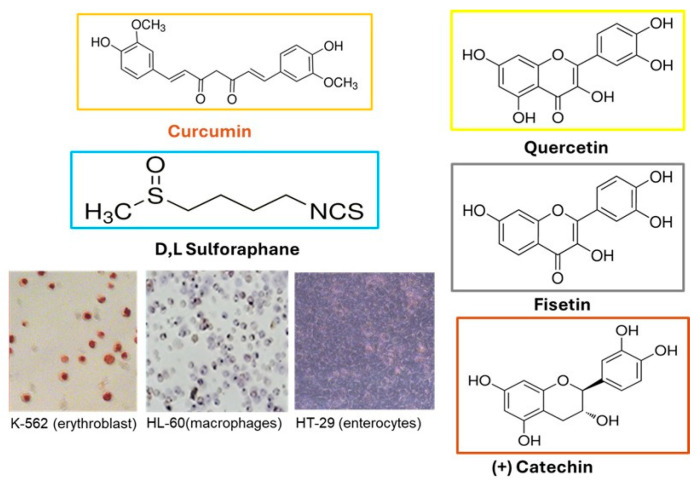
Chemical structure of selected phytochemicals (https://www.sigmaaldrich.com/) and micrograph (obtained after differentiation at 400× magnification with the inverted microscope Axiovert 200 Zeiss) of cellular models used to screen their chemopreventive effects, which will be described in the study.

Figure 1 reports the molecular structures of different phytochemicals and the human cell lines used to test their chemopreventive effects.

### 3.2. Curcumin at Low Doses Protects K-562 Differentiated Cells from Oxidative Stress by Activating the HO-1 Transcript and Protein

**Figure 2:**
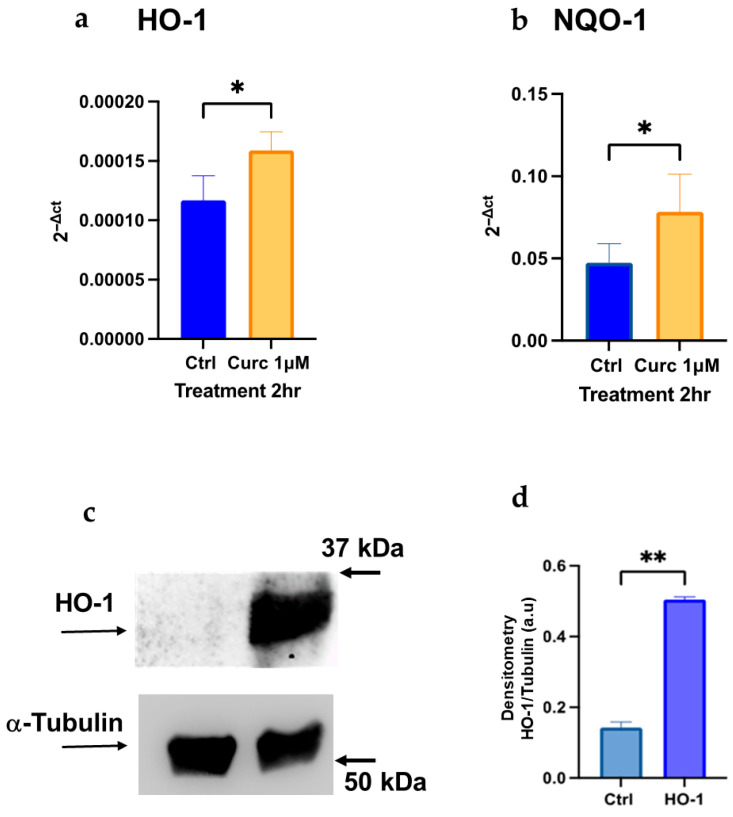
Curcumin at a low dose induces expression of the Nrf2-dependent antioxidant enzymes in K-562 differentiated cells. qRT-PCR results of HO-1 (**a**) and NQO-1 (**b**) after incubation of K-562 cells with Curc 1 μM for 2 h. (**c**) Immunoblot analysis of HO-1 expression in K-562 cells after 16 h incubation with Curc 1 μM. Quantification of protein bands is shown in the histograms (**d**) and was achieved by Image Lab software (Biorad) by measuring the ratio of HO-1/α-Tubulin. Bar graph indicates the mean of *n* = 3 experiments in duplicate ± SD (**a**,**b**) and *n* = 2 experiments (**d**). The original Western blot images are shown in Figure S3. Asterisks indicate statistical significance after the *t*-test analysis: * indicates *p* < 0.05; ** indicates *p* < 0.01.

We previously demonstrated that Curc at 1 μM exhibits antioxidant effects, activating NQO-1 and HO-1 transcription and protein expression in HL-60 differentiated cells [17]. Here, we confirm these data, showing that Curc can also induce HO-1 and NQO-1 at the mRNA level in differentiated K-562 cells, which are committed to the erythroid lineage after treatment with 50 μM resveratrol [28]. For HO-1, we also evaluated its expression for the first time in these cells, at both the mRNA and protein levels (Figure 2).

### 3.3. Opposite Effects of Low and High Doses of Sulforaphane on Oxidative Stress in K-562 Differentiated Cells via Nrf2/ARE Pathway Activation

**Figure 3:**
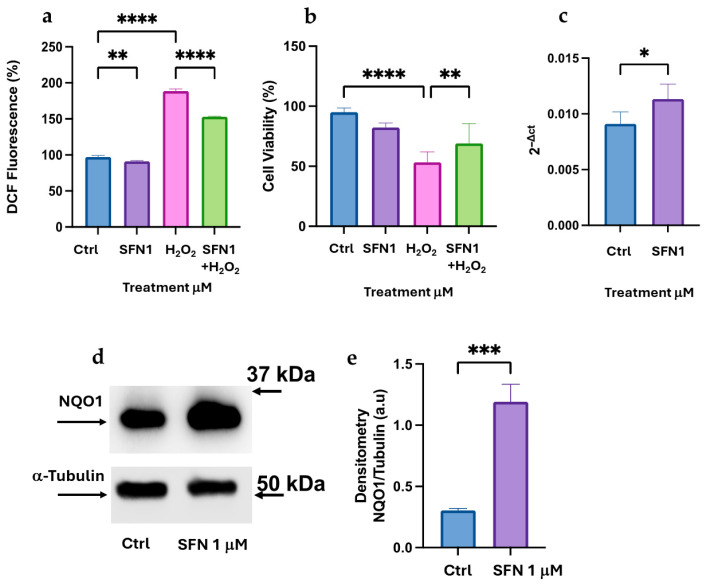
Sulforaphane at low doses protects K-562 differentiated cells from H_2_O_2_ toxicity and induces the expression of the NQO-1. (**a**) Intracellular peroxide analysis with DCF-DA probe after overnight incubation with 1 μM SFN in differentiated K-562 cells stressed with 50 μM H_2_O_2_ for 30 min. (**b**) Cell viability assay after SFN treatment as in (**a**). Expression of NQO-1 measured by qRT-PCR (**c**) and Immunoblot analysis (**d**,**e**) in differentiated K-562 cells after overnight incubation with SFN 1 μM. Quantification of protein bands is shown in the histograms (**e**) and was achieved by Image Lab software (Biorad) by measuring the ratio of NQO-1/α-Tubulin. Bar graph indicates the mean of *n* = 3 experiments in duplicate ± SD (**a**–**c**) and *n* = 2 experiments (**e**). The original Western blot images are shown in Supplementary Figure S5. Asterisks indicate statistical significance after the ANOVA (multiple comparison) or Student’s *t*-test (two-group of data) analysis: * = *p* < 0.05; ** = *p* < 0.01, *** = *p* < 0.001; **** = *p* < 0.0001.

**Figure 4:**
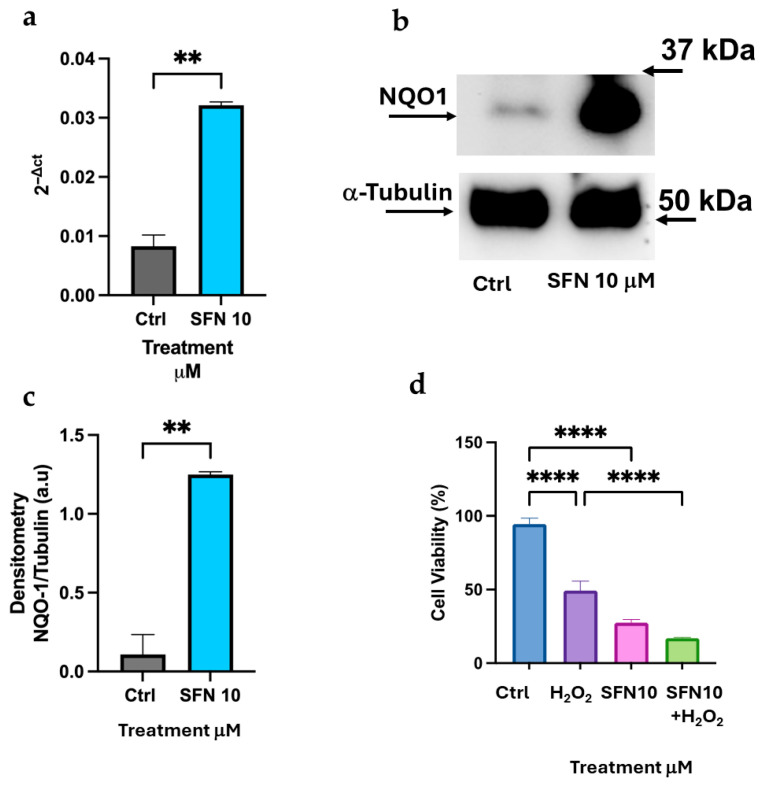
Higher expression of NQO-1 at the mRNA and protein levels is not associated with a protective effect of SFN in differentiated K-562. Expression of NQO-1 measured by qRT-PCR (**a**) and Immunoblot (**b**,**c**) in differentiated K-562 cells after 16 h of treatment with 10 μM SFN. (**d**) Cell viability assay after SFN 16 h incubation of differentiated K-562 cells, then stressed for 24 h with 50 μM H_2_O_2_. Quantification of protein bands is shown in the histogram (**c**) and was performed using Image Lab software by measuring the NQO-1/α-Tubulin ratio. Bar graph indicates the mean of *n* = 2 experiments in triplicate ± SD (**a**–**d**) and *n* = 2 experiments (**c**). The original Western blot images are shown in Supplementary Figure S4. Asterisks indicate statistical significance after ANOVA (multiple comparisons) or Student’s *t*-test (two-group data): ** = *p* < 0.01, **** = *p* < 0.0001.

We compared the effect of 1 μM Curc in K-562 cells with SFN, a well-known Nrf2 activator. SFN at 1 μM exhibits antioxidant effects on K-562 differentiated cells, resulting in less than 30% peroxide production and a significant cell viability rescue after oxidative stress (Figure 3a,b). SFN activates NQO-1 transcription and protein expression in K-562 differentiated cells (Figure 3c–e), suggesting that SFN replicates the effect of Curc on the same cellular model [17]. However, when higher concentrations of SFN (10 μM) were applied to differentiated K-562 cells, we confirmed a significant expression of both mRNA and protein levels of NQO-1 (Figure 4a–c). Importantly, in these experimental conditions, SFN was cytotoxic (>50%) (Figure 4d). Similarly, at 10 μM, SFN was not able to protect differentiated K-562 cells from oxidative damage caused by H_2_O_2_ addition (Figure 4d). We concluded that the elevated expression of NQO-1, both at the mRNA level (over 3-fold compared to untreated cells) and the protein level (over 10-fold compared to control), was unable to counteract the cytotoxicity of SFN at high concentrations. These findings indicate that high-dose SFN significantly activates NQO-1; however, it does not confer cytoprotection due to its intrinsic cytotoxicity at elevated doses.

### 3.4. Quercetin and Other Flavonoids Applied at a Low Dose Are Not Efficacious in K-562 Differentiated Cells

**Figure 5:**
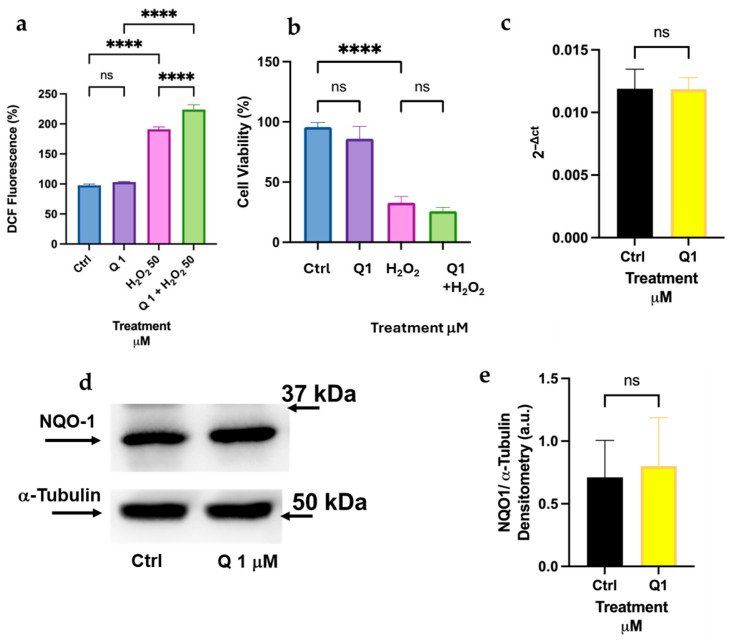
Quercetin is unable to protect differentiated K-562 cells from oxidative stress. (**a**) Levels of intracellular peroxides after Q treatment for 16 h of differentiated K-562 cells, followed by the addition of 50 μM H_2_O_2_ for 30 min. (**b**) Cell viability assay after Q treatment for 16 h, followed by the addition of 50 μM H_2_O_2_ for 24 h. (**c**) Expression by qRT-PCR of NQO-1 after 16 h incubation with 1 μM Q. (**d**,**e**) Immunoblot analysis of NQO-1 expression in differentiated K-562 cells after treatment with 1 μM Q for 16 h. Quantification of protein bands is shown in the histograms (**e**) and was performed using Image Lab software by measuring the NQO-1/α-Tubulin ratio. The original Western blot images are shown in Supplementary Figure S6. Asterisks indicate statistical significance after the ANOVA (multiple comparison) or Student’s *t*-test (two-group of data) analysis: **** = *p* < 0.0001. ns: not significant.

To continue our screening, we verified if another well-known natural antioxidant, quercetin (Q), could protect differentiated K-562 cells from oxidative stress. We preincubated cells with 1 μM Q for 16 h, then removed the culture medium, and stressed cells with 50 μM H_2_O_2_ for 30 min to measure intracellular peroxide levels using the DCF-DA assay (Figure 5a). We also measured changes in cell viability 24 h after treatment with 50 μM H_2_O_2_ (Figure 5b). In both cases, we did not detect any protection exerted by Q at the low concentration applied. Accordingly, we did not measure significant differences in the expression of NQO-1 (mRNA and protein, Figure 5c–e).

**Figure 6:**
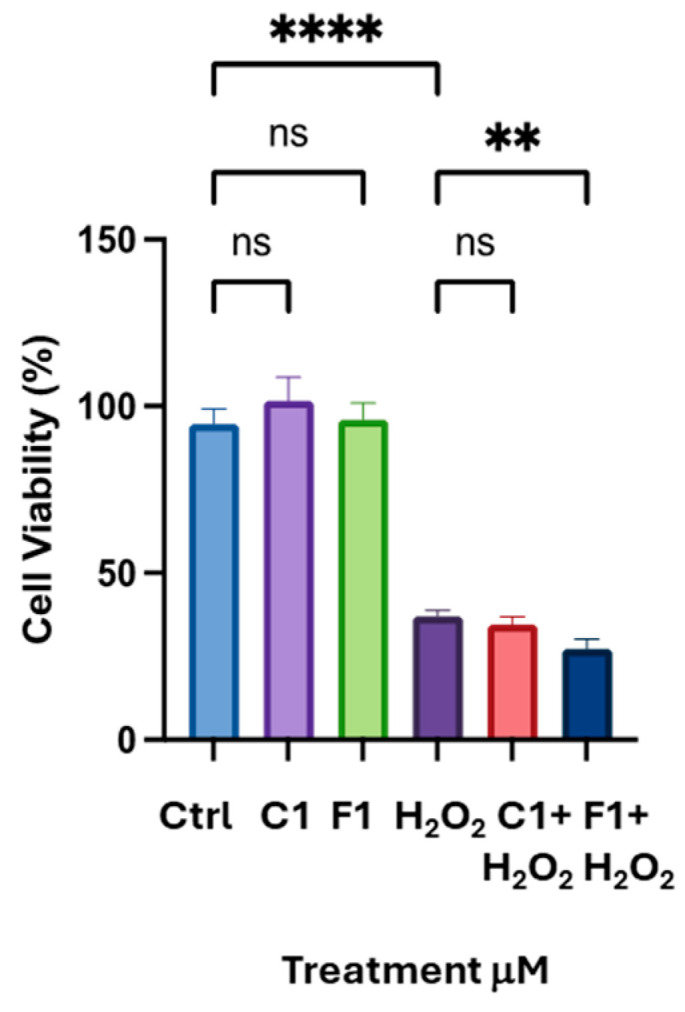
Fisetin and Catechin, at low doses, are unable to protect K-562 cells from oxidative stress. Cell viability assay after 16 h of incubation of differentiated K-562 cells with F and C, followed by 24 h of incubation with 50 μM H_2_O_2_. Bar graph indicates the mean of *n* = 2 experiments in quadruplicate ± SD. Asterisks indicate statistical significance after ANOVA statistical Test: ** *p* < 0.01; **** *p* < 0.0001; ns: not significant.

When two other flavonoids structurally related to Q were tested, we obtained similar results. Fisetin (F) has four hydroxyl moieties in the C and A rings, and (+) Catechin (C) shows no chetone group in the B ring (Figure 1). The results of the cell viability assay in Figure 6 demonstrate that neither F nor C can protect K-562 cells from H_2_O_2_-induced oxidative stress. We did not use higher concentrations of flavonoids due to concerns about toxicity in these cells. Thus, quercetin and structurally related flavonoids are ineffective in the K-562 model at physiologically relevant doses.

### 3.5. Pre-Incubation with Curcumin and Flavonoid Quercetin and Fisetin, but Not Sulforaphane, Protects HL-60 Differentiated Cells from H_2_O_2_-Induced Toxicity

**Figure 7:**
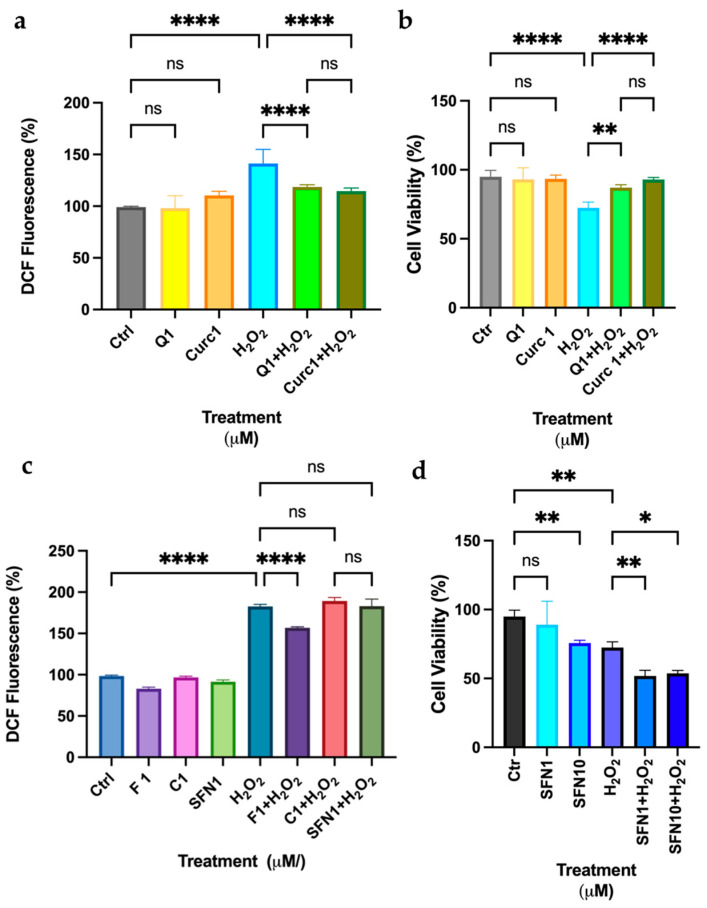
Quercetin, Fisetin, and Curcumin at low doses have a comparable effect in protecting HL-60 cells from oxidative stress. (**a**–**c**) Intracellular peroxide levels were measured using the DCF-DA fluorescent probe after differentiated HL-60 cells were incubated for 2 h with the indicated polyphenols (1 µM), followed by a 30 min exposure to H_2_O_2_ (10 µM). (**b**–**d**) Cell viability was assessed after differentiated HL-60 cells were incubated with the indicated phytochemicals for 2 h, followed by a 24 h exposure to 10 µM H_2_O_2_. Bars indicate the mean of *n* = 2 experiments in quadruplicate ± SD in (**a**–**d**). Asterisks indicate statistical significance after the ANOVA test. * *p* < 0.05, ** *p* < 0.01, **** *p* < 0.0001. ns: not significant.

Based on the previous findings, we investigated whether the selective effects of phytochemicals at low doses on differentiated K-562 cells could be reproduced in another myeloid-derived model, the HL-60 cell line, which differentiates into the myelomonocytic lineage upon vitamin D (VD) treatment [17]. HL-60 cells represent a suitable model for studying the protective efficacy of natural biactives because they exhibit the peculiar characteristic of differentiating towards the granulocytic/macrophage lineage after the addition of vitamin D_3_ analogue, EB1089, or the precursor of vitamin A, all-trans-retinoic acid (ATRA), resembling their normal counterpart [26]. In a previous study, we demonstrated that Curc at low doses could efficiently protect differentiated HL-60 from Cadmium and H_2_O_2_-induced oxidative stress [17]. As shown in Figure 7a, Q and Curc are equally effective in reducing intracellular peroxides (<30%) after stressing HL-60 cells with H_2_O_2_ and, comparably, safeguarding cell viability after 24 h from the oxidative damage (<20%) (Figure 7b).

We also tested SFN and other flavonoids, such as F and C, in the same experimental model. Figure 7c demonstrates that only F can reproduce Q and Curc effects in differentiated HL-60. Surprisingly, SFN at high doses (10 μM) and the combination with low doses and H_2_O_2_ were highly cytotoxic in HL-60 cells, unlike in the erythroblastoid K-562 model (Figure 7c,d).

### 3.6. Sulforaphane, Curcumin, and Quercetin Interfere with the Nrf2-Dependent Pathways in Differentiated HL-60 Cells

**Figure 8:**
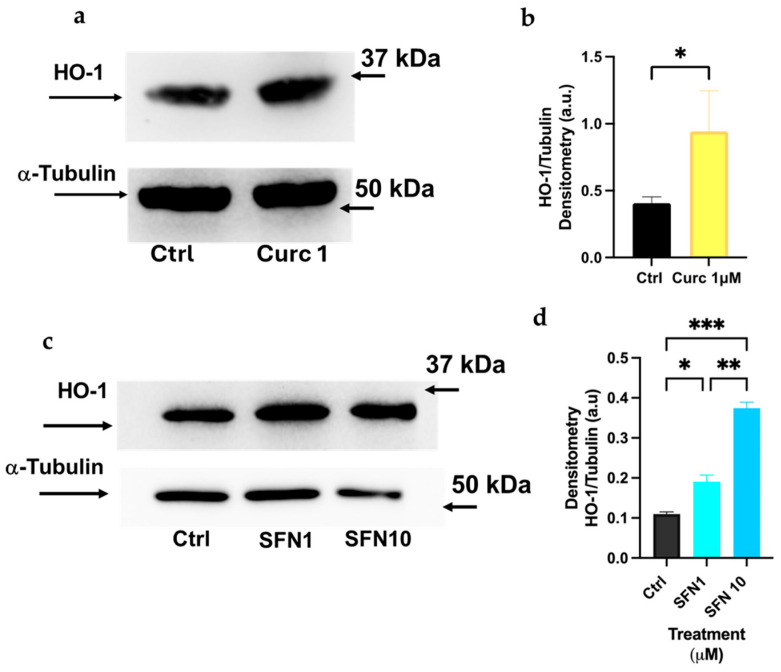
Sulforaphane and Curcumin at low doses exhibit significant efficacy in inducing HO-1 expression in differentiated HL-60 cells. (**a**,**b**) Immunoblot and densitometric analysis of HO-1 expression after 2 h incubation of differentiated HL-60 cells with 1 μM Curc. (**c**,**d**) Immunoblot and densitometric analysis of differentiated HL-60 cells treated overnight with SNF 1 or 10 μM. Quantification of protein bands is shown in the histograms (**b**,**d**) and was achieved by Image Lab software by measuring the ratio of HO-1/α-Tubulin. Bars indicate the mean of *n* = 2 experiments ± SD (**b**–**d**). The original Western blot images are shown in Supplementary Figure S8a–c. Asterisks indicate statistical significance after Student’s *t*-test or ANOVA test. * *p* < 0.05, ** *p* < 0.01, *** *p* < 0.001.

In the following experiments, we compared SFN, Curc, and Q in terms of their ability to activate Nrf2-dependent pathways, specifically via increasing the expression of HO-1 and NQO-1 enzymes in differentiated HL-60 cells. In Figure 8a,b, we demonstrated that Curc and SFN at 1 μM concentrations are equally effective in upregulating HO-1 expression (>2-fold with respect to untreated). SFN at 10 μM concentration is more efficacious in raising HO-1 expression, as the protein level is four times higher compared to control cells (Figure 8c,d).

**Figure 9:**
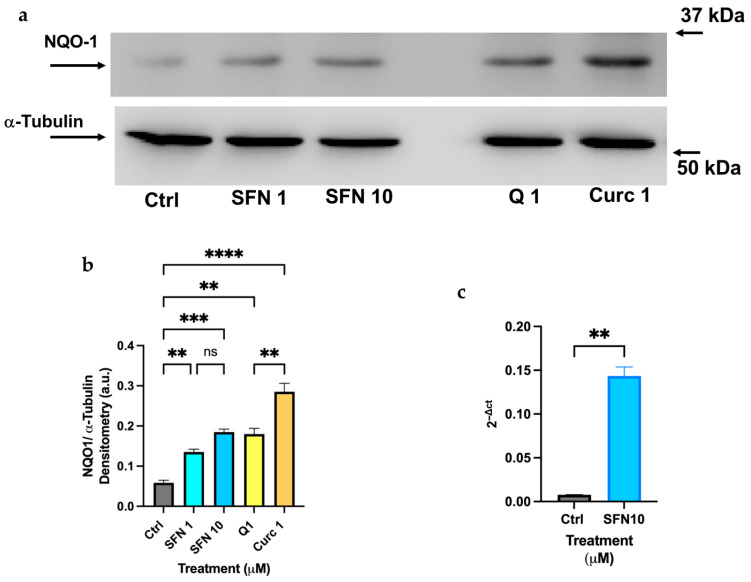
Quercetin, Sulforaphane, and Curcumin at low doses exhibit significant efficacy in inducing NQO-1 expression in HL-60 cells. (**a**,**b**) Immunoblot analysis of NQO-1 expression in HL-60 cells after 2 h of incubation with indicated compounds (1–10 μM), (**c**) qRT-PCR results of NQO-1 mRNA expression after 16 h of incubation with 10 μM SFN. Quantification of protein bands is shown in the histogram (**b**) and was performed using Image Lab software by measuring the NQO-1/α-Tubulin ratio. Bars indicate the mean of *n* = 2 experiments ± SD in (**b**) and *n* = 3 experiments in duplicate ± SD (**c**). The original Western blot images are shown in Supplementary Figure S7. Asterisks indicate statistical significance after the ANOVA (multiple comparison) or Student’s *t*-test (two-group of data) analysis: ** = *p* < 0.01, *** *p* < 0.001; **** = *p* < 0.0001. ns: not significant.

Figure 9a shows the results of the Immunoblot analysis obtained in differentiated HL-60 cells after the addition of the indicated concentrations of phytochemicals (Curc, Q, and SFN), resulting in the increased expression of NQO-1 antioxidant factor. For SFN, the upregulation of NQO-1 was also assessed by measuring its mRNA level after treatment.

The experimental results confirm that Curc exhibits the strongest ability to upregulate NQO-1 at the protein level, showing more than a 3-fold increase compared with the control. However, both Q and SFN at the same concentration (1 μM) also significantly increased NQO-1 protein levels by more than 2-fold (Figure 9a,b). Furthermore, treatment with SFN at 10 μM resulted in a 10-fold increase in NQO-1 transcript levels in HL-60 cells after overnight incubation in culture medium, consistent with findings from previous studies.

### 3.7. Antioxidant Effects of Quercetin and Curcumin at Low Doses on Differentiated HT-29 Cells

**Figure 10:**
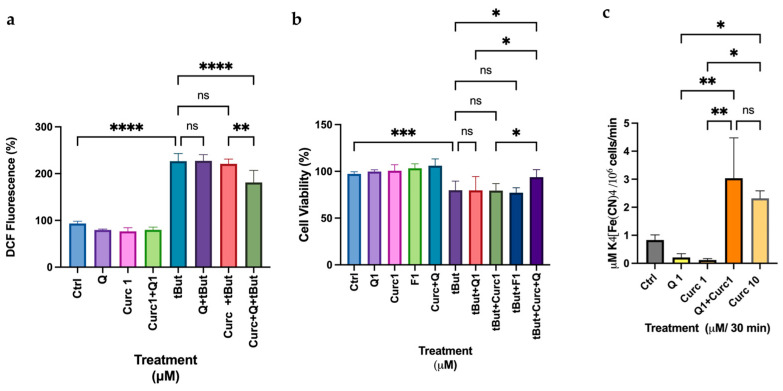
Quercetin, when combined with low doses of Curcumin, protects differentiated HT-29 cells from oxidative stress by activating the Plasma Redox Membrane System. (**a**) Intracellular peroxides measurement with DCF-DA fluorescent probe after 2 h incubation of differentiated HT-29 cells with indicated polyphenols. (**b**) Crystal Violet assay after incubation for 2 h with selected polyphenols at 1 μM or co-incubation of Curc+Q. Cells were washed and stressed, where indicated, with t-But (0.5 mM). (**c**) Plasma Membrane Reducing System Enzymatic Assay (PMRS) of HT29 differentiated cells after 30 min of treatment with the indicated molecules. Bars indicate the mean of *n* = 2 experiments in quadruplicate ± SD (**a**–**c**). Asterisks indicate statistical significance after the ANOVA test. * *p* < 0.05; ** *p* < 0.01, *** *p* < 0.001; **** *p* < 0.0001. ns: not significant.

In addition to the cellular model employed above, we reasoned that it would be of interest to investigate the protective effects of the selected phytochemicals in a cellular model of intestinal epithelial cells, which can mimic the absorption of these compounds following the consumption of plant-based foods or nutraceuticals. For this reason, we used the HT-29 cell line differentiated with sodium butyrate (NaBt) as a model of a mature enterocyte with a brush border, expressing high levels of specific markers (e.g., alkaline phosphatase, ALP; a brush border hydrolytic enzyme playing an essential role in the maintenance of intestinal microbial homeostasis and intestinal barrier function through its ability to dephosphorylate lipopolysaccharide, Figure S1) [32]. Figure 10a shows the results of intracellular peroxide measurement using the DCF-DA method. Neither Curc nor Q at 1 μM dose can protect these cells from oxidative stress induced by t-But; however, when Curc and Q were combined (1 μM each), a low but significant protective effect was observed in lowering peroxides after treatment (about 20% protection). This result was supported by the cell viability measurement (Figure 10b), which showed that the combination Q+Curc was more efficient than the single molecules in reducing t-But cytotoxicity. Since the Immunoblot analysis of NQO-1 and HO-1 expression indicated that these enzymes dependent from Nrf2 activity were not involved in the protective effect observed, we evaluated the activity of a multienzyme complex on the cell membrane, the Plasma Membrane Reducing System (PMRS) (Figure 10c), an essential system to safeguard cellular homeostasis after an oxidative external stress. The results indicate that only the combined treatment of Cur plus Q significantly enhanced PMRS activity (more than three-fold) after 30 min of treatment. Finally, SFN, tested at low and high concentrations (1–10 M), was ineffective to counteract oxidative stress induced by t-But in this model (Figure S2). These findings suggest a cell-type-specific response to phytochemicals at low doses, with HT-29 cells relying more on membrane-associated redox systems than on canonical Nrf2 signaling.

## 4. Discussion

The present study assessed the efficacy of various phytochemicals in targeting the Nrf2-related pathway (NQO1-HO-1) across different cellular models, thereby conferring protection against oxidative damage. The results of this screening are summarized in Table 1.

In K-562 cells, only SFN at a low dose can mimic the protective effect of Curc (Figure 2 and Figure 3); in fact, at higher doses (10 μM), SFN showed a toxic effect in presence or absence of oxidative stress (Figure 4d), confirmed by a previous study, and despite a higher expression of NQO-1 mRNA and protein observed (Figure 4a–c) [37]. This behavior resembles the classical biphasic curve of a hormetic response, where low doses of an agent are protective against an exogenous stress. In contrast, higher doses are cytotoxic, despite the higher expression of a “protective” enzyme, e.g., NQO-1, whose high levels represent the attempt of the cell to counteract a death stimulus [11,12,38]. This biphasic response is consistent with classical hormesis, where a mild oxidative stimulus activates cytoprotective pathways, whereas higher concentrations exceed adaptive capacity and trigger cell death. In fact, we observed that SFN exhibited a similar response pattern in differentiated HL-60 cells. In this context, SFN effectively induced the expression of Nrf2-dependent effectors, such as NQO-1 and HO-1 (Figure 8 and Figure 9). However, it also displayed marked cytotoxicity at the concentrations tested (10 μM alone and 1–10 µM in combination with H_2_O_2_; Figure 7d), likely reflecting the higher sensitivity of these cells to oxidative stress. This notion is supported by the lower concentration of H_2_O_2_ required to elicit toxicity in HL-60 cells (10 μM) compared with K-562 cells (50 μM). It is conceivable that even a minimal dose of SFN, which is not intrinsically cytotoxic, induces Nrf2-dependent transcripts, but also adversely affects specific and unknown cytoprotective pathways when metabolized within HL-60 cells and subsequently subjected to oxidative stress via H_2_O_2_. In other words, the eustress induced by SFN in HL-60 cells should be reached with a very low concentration of SFN (<1 μM). Nevertheless, administering the same SFN dose to HL-60 and K-562 cells enables us to validate these intriguing opposing effects within these experimental models.

However, the use of a very low dose of SFN in HL-60 differentiated cells may be closer to the realistic plasma concentration of the molecule, assuming SFN is consumed with food [39,40]. In future studies, HL-60 cells, a well-established preclinical model of acute myeloid leukemia, could be compared with their differentiated counterparts used in this study at the same low SFN concentrations (0.1–0.5 μM) to better distinguish between chemopreventive and chemotherapeutic effects [41]. Interestingly, a proof-of-concept study demonstrated that SFN intake did not reduce neutrophilic airway inflammation nor enhance redox markers in peripheral blood mononuclear cells (PBMCs), thereby confirming, in vivo, the ineffectiveness of SFN in the preclinical model of differentiated HL-60 employed in this investigation [42].

Curiously, results from this screening between differentiated K-562 and HL-60 cells also show different behavior with respect to Q and related flavonoids, such as F and C. For erythroblast cells, neither Q nor F nor C protected against H_2_O_2_-induced cytotoxic stress (Figure 5). Accordingly, Q was unable to upregulate NQO-1 expression at either the transcript or protein level (Figure 5c–e). In differentiated HL-60, Q and F showed comparable antioxidant activity, whereas C was ineffective in protecting them from oxidative stress (Figure 7a–c). Unlike K-562, HL-60 cells showed a distinct ability to upregulate NQO-1 protein expression after incubation with low doses of Q (Figure 9a,b) [22]. These surprisingly different results, obtained with the same molecules and concentrations, reveal that Q and other flavonoids of similar structure may be metabolized differently in different tissues within the organism after dietary absorption [6]. This hypothesis is difficult to test in vivo; however, this can be readily examined in vitro. For instance, as demonstrated in the previous publication, we were able to measure free curcumin inside HL-60 cells after only 5 min of adding the polyphenol to the cell culture medium [17]. In continuation of this study, it is feasible to quantify curcumin and its intracellular metabolites—such as oxidized curcumin and degradation products like vanillic and ferulic acid—in myeloid and intestinal cellular models. This measurement will facilitate the verification and comparison of intracellular concentrations of curcumin and its active metabolites following low-dose administration. A limitation of this study is the lack of intracellular analysis of these metabolites in the three cellular models; a future manuscript will address this key point. It is crucial to confirm the presence of various metabolite forms—such as methylated, glucuronidated, or oxidized—of the same molecule within these models. However, implementing these measures may be difficult due to the absence of specific standards for some metabolites, such as oxidized forms and degraded products, which are still unknown.

Future research, which extends beyond the scope of this manuscript, could expand to incorporate other phytochemicals, including Q, F, C, and SFN, along with their respective metabolites, provided that specific standards are available. This would facilitate the evaluation of their bioavailability in HL-60, K-562, and HT-29 preclinical models. Such comparative analyses may elucidate the differential outcomes observed during preliminary screening.

Notably, we observed that C was entirely ineffective as an antioxidant at low doses across all cellular models examined in this study. (+) Catechin was selected due to its structural similarity to Q and F (Figure 1). However, it lacks a ketone group at position 4 of the C ring. This omission may be a critical factor affecting its bioavailability and bioactivity, as evidenced by our previous research, in which we compared its antioxidant activity in leukemic cells with that of quercetin [43]. The study demonstrated that a concentration of 100 μM of Catechin could significantly reduce intracellular peroxides (<40%), whereas achieving the same effect with Quercetin (<60%) required only 50 μM. Lastly, an in vivo study in rats indicated that dietary catechin was ineffective at increasing NQO-1 activity, even at a high dose (2 g/kg diet) [44].

Specifically, our data substantiate that the realistic nutritional antioxidant effect of phytochemicals is achieved through the paradoxical oxidative activation of the Nrf2 signaling pathway, which supports the maintenance of protective oxidoreductases, but this effect is tissue-specific [20].

In fact, the intriguing data obtained from the screening substantiated the selectivity of various polyphenols by evaluating low doses of Q and Curc in a model of mature enterocytes, represented by differentiated HT-29 cells. Neither Curc nor Q demonstrated the ability to protect these cells from oxidative stress; however, when combined, these compounds offered modest yet statistically significant protection (Figure 10b,c). This outcome can be attributed to the hypothesis that**,** within the human body, the intestine functions as a physical barrier against exogenous, potentially toxic substances present in the diet. The lack of a protective effect from individual phytochemicals (Curc or Q) or the significant antioxidant and protective activity observed with their mixture may be due to unknown metabolic pathways or absorption mechanisms associated with a unique mode of action that warrants further investigation. One of these mechanisms could be related to another type of adaptive response not related to the Nrf2 system: we showed that PMRS is clearly involved in the protective mechanism, confirming the ability of a mixture of plant polyphenols as electron donors for the PMRS not only on the erythrocyte membrane but also at the intestinal level [23,45]. Clearly, this adaptive response may also be relevant in other cellular models, such as the myeloid lineage, as we intend to demonstrate in a future publication.

From an applicative point of view, this research could shed light on the nutrition ‘dark matter’ represented by dietary phytochemicals and be helpful in the rational and optimized formulation of nutraceuticals/functional foods. In the following studies, we will explore how these compounds can be further modified or encapsulated in nanocarriers to achieve more substantial intracellular uptake and stability, leading, hopefully, to the discovery of more efficacious nutraceuticals or mixed phytochemicals for use as supplements in human disease prevention.

## 5. Conclusions

Our investigation demonstrates, for the first time in experimental cell models, that not all phytochemicals are effective antioxidants at low doses. Low-dose phytochemicals activate the Nrf2 axis via a controlled oxidative mechanism, but the cellular outcome is tissue-specific. In K-562 differentiated erythroblast cells, Cur and sulforaphane SFN at 1 μM concentration effectively protect cells from oxidative stress, with Nrf2 contributing to this protective mechanism. However, SFN is toxic at high doses in these cells, and flavonoids with similar chemical structures, Q, C, and F, show no protective effect. In HL-60 granulocytic-differentiated cells, Cur and structurally related flavonoids (Q, F) exhibit comparable antioxidant activity at low doses (1 μM), whereas SFN is toxic at both low and high doses in association with H_2_O_2_, despite increased expression of HO-1 and NQO-1 proteins. In HT-29 enterocytic-differentiated cells, neither Cur nor Q is effective at low doses; however, their combination confers protection against t-butyl hydroperoxide-induced oxidative stress by activating PMRS. Overall, these findings indicate that the antioxidant efficacy of phytochemicals at low doses does not always correlate with elevated expression of NQO-1 or HO-1 proteins or transcripts in myeloid cells.

The present manuscript confirms for the first time that the health benefits of low doses of phytochemicals are linked to a hormetic response known as eustress, which protects cells against external oxidative stress, a process often linked to chronic-degenerative diseases associated with aging.

## Figures and Tables

**Table 1:** Resume of the screening of selected phytochemicals in human preclinical cell models.

Phytochemical	Cellular Model ^1^	Protective Effect ^2^
Curcumin [35]	Erythroblasts (K-562)	Verified [17]
Curcumin [35]	Macrophages (HL-60)	Verified [17]
Curcumin [36]	Enterocytes (HT-29)	Not present
Quercetin	Erythroblasts (K-562)	Not present
Quercetin	Macrophages (HL-60)	Verified
Quercetin	Enterocytes (HT-29)	Not Present
Sulforaphane	Macrophages (HL-60)	Not Present
Sulforaphane	Erythroblasts (K-562)	Verified
Sulforaphane	Enterocytes (HT-29)	Not Present
Fisetin	Macrophages (HL-60)	Verified
Fisetin	Erythroblasts (K-562)	Not Present
Fisetin	Enterocytes (HT-29)	Not Present
Catechin	Macrophages (HL-60)	Not Present
Catechin	Erythroblasts (K-562)	Not Present

^1^ K-562 cells were treated for 24 h with 50 μM Resveratrol to differentiate cells towards erythroblast lineage; HL-60 were treated for 72 h with VD analogue EB1089 100 nM to obtain a differentiation towards a macrophage lineage; HT-29 cells were differentiated into mature enterocytes by adding NaBt 4 mM to the medium for 72 h. ^2^ Evaluated at a concentration of 1 μM against H_2_O_2_ for HL-60 and K-562 or t-butyl induced toxicity for HT-29.

## Data Availability

The original contributions presented in this study are included in the article/Supplementary Material. Further inquiries can be directed to the corresponding author.

## Supplementary Materials

The following supporting information can be downloaded at: https://www.mdpi.com/article/10.3390/biom16020191/s1. Figure S1: Alkaline phosphatase activity (ALP) in HT-29; Figure S2: Sulforaphane at low and high doses does not protect differentiated HT-29 cells from oxidative stress. HT-29; Table S1: qRT-PCR primers, Supplementary Figures S3–S8 are relative to the original WB images.

## Abbreviations

The following abbreviations are used in this manuscript:

NDM	Nutrition dark matter
NQO-1	NADPH Quinone Oxidoreductase-1
HO-1	Heme Oxygenase-1
Nrf2	Nuclear factor erythroid 2 (NF-E2)-related factor 2
Curc	Curcumin
SFN	Sulforaphane
Q	Quercetin
C	Catechin
F	Fisetin
ALP	Alkaline Phosphatase
t-But	t-Butyl-hydroperoxide
PMRS	Plasma Membrane Reducing System
CM-DCF-DA	Chloromethyl derivative of 2,7-Dichlorodihydrofluorescein diacetate
VD	Vitamin D
NaBt	Sodium Butyrate
ATRA	All Trans Retinoic Acid
GAPDH	GlycerAldehyde-3-Phosphate DeHydrogenase
