# Supplementary material for: Phytochemicals Possess Selective Chemopreventive Mechanisms That Safeguard Human Cells from Oxidative Toxicity
Source: Biomolecules. 2026 Jan 27;16(2):191. doi: 10.3390/biom16020191 (PMC12937693; doi:10.3390/biom16020191)

S1

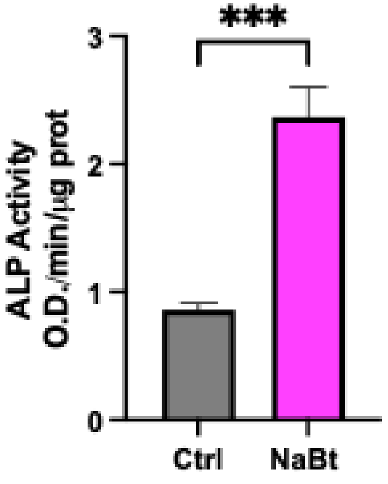

Figure S1. Alkaline phosphatase activity (ALP) in HT-29
Enzymatic activity of ALP in HT29 cells after treatment with sodium butyrate (NaBt) 4 mM for 72 h. Bars indicate the mean of two experiments in triplicate ± SD. Asterisks indicate statistical significance after T-test Students test. ***p<0.001.

S2

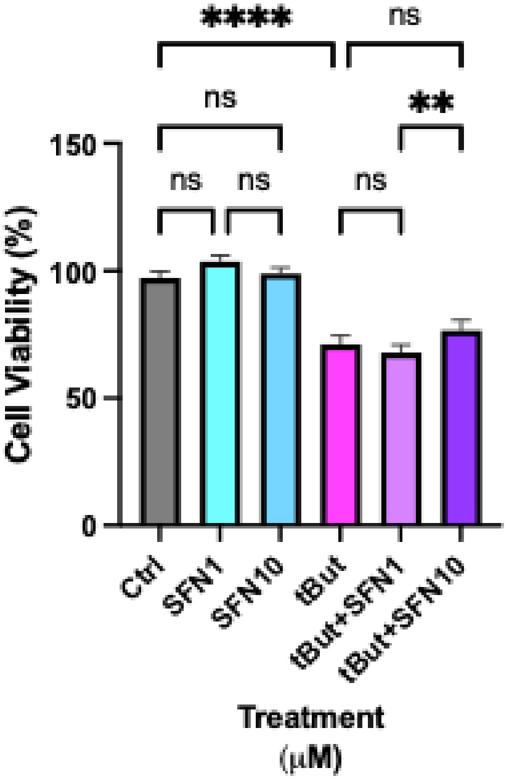

Figure S2. SFN at low and high doses does not protect differentiated HT-29 cells from oxidative stress. HT-29 differentiated cells were incubated with SFN for 2 hours, then it was removed from the cell culture medium, and where indicated, replaced with tBut 0.5 mM to induce oxidative stress. Cell viability was evaluated with Crystal Violet assay after 24 hours. Bars indicate the mean of two experiments in quadruplicate ± SD. Asterisks indicate statistical significance after the ANOVA test. **p<0.01, ****p<0.0001.

Table S1 qRT-PCR primers

Gene	Primer F	Primer R
**NQO1**	GGATTGGACCGAGCTGGAA	AATTGCAGTGAAGATGAAGGCAAC
**HO**	CCAGGCAGAGAATGCTGAGTTC	AAGACTGGGCTCTCCTTGTTGC
**GAPDH**	CCACTAGGCGCTCACTGTTCT	GCGAACTCACCCGTTGACT

Fig. Supp 3 C Tubulin-HO-1 K-562 Curc

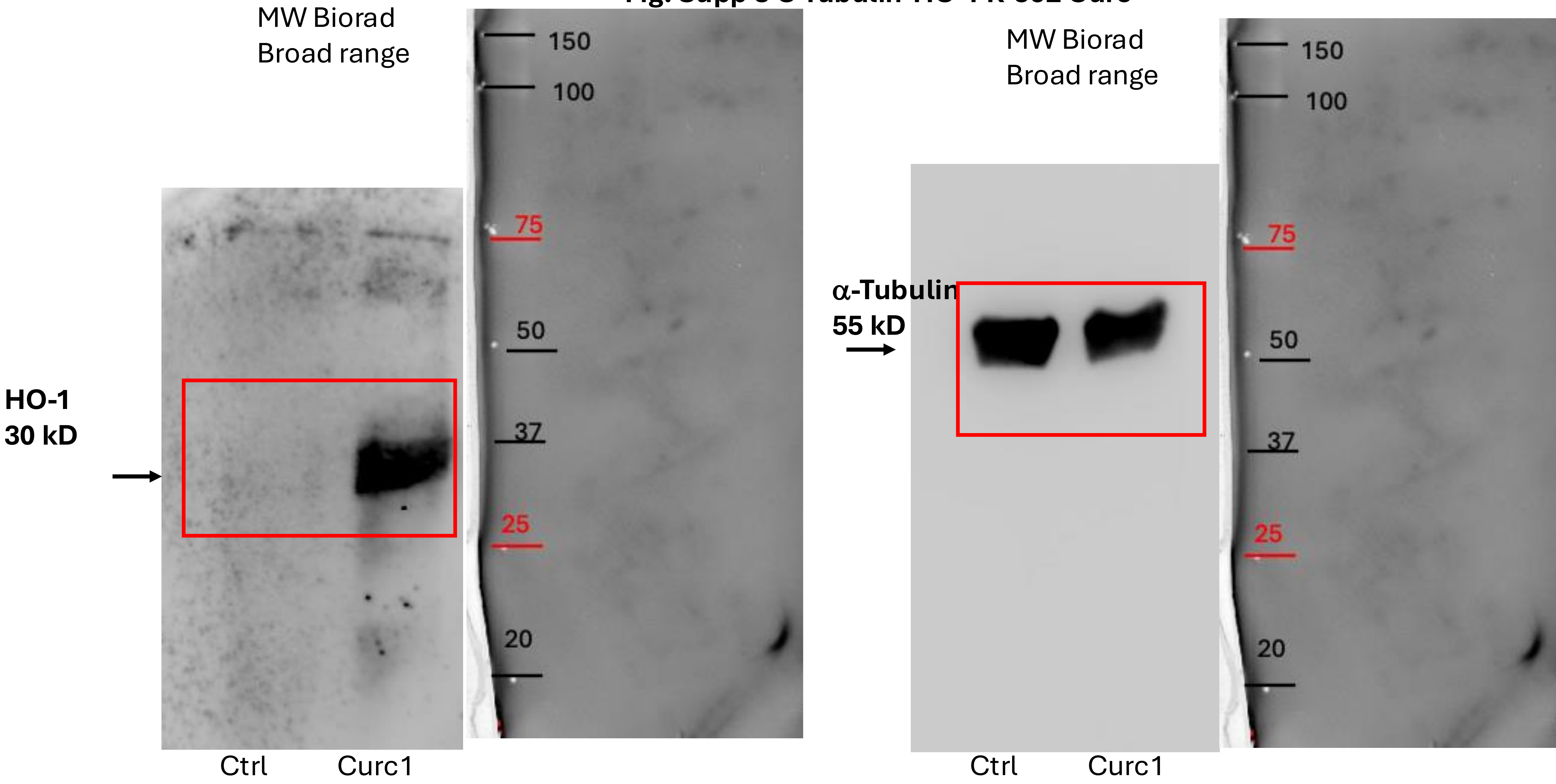

Fig. Supp 4 B NQO1-Tubulin K-562 SFN 10

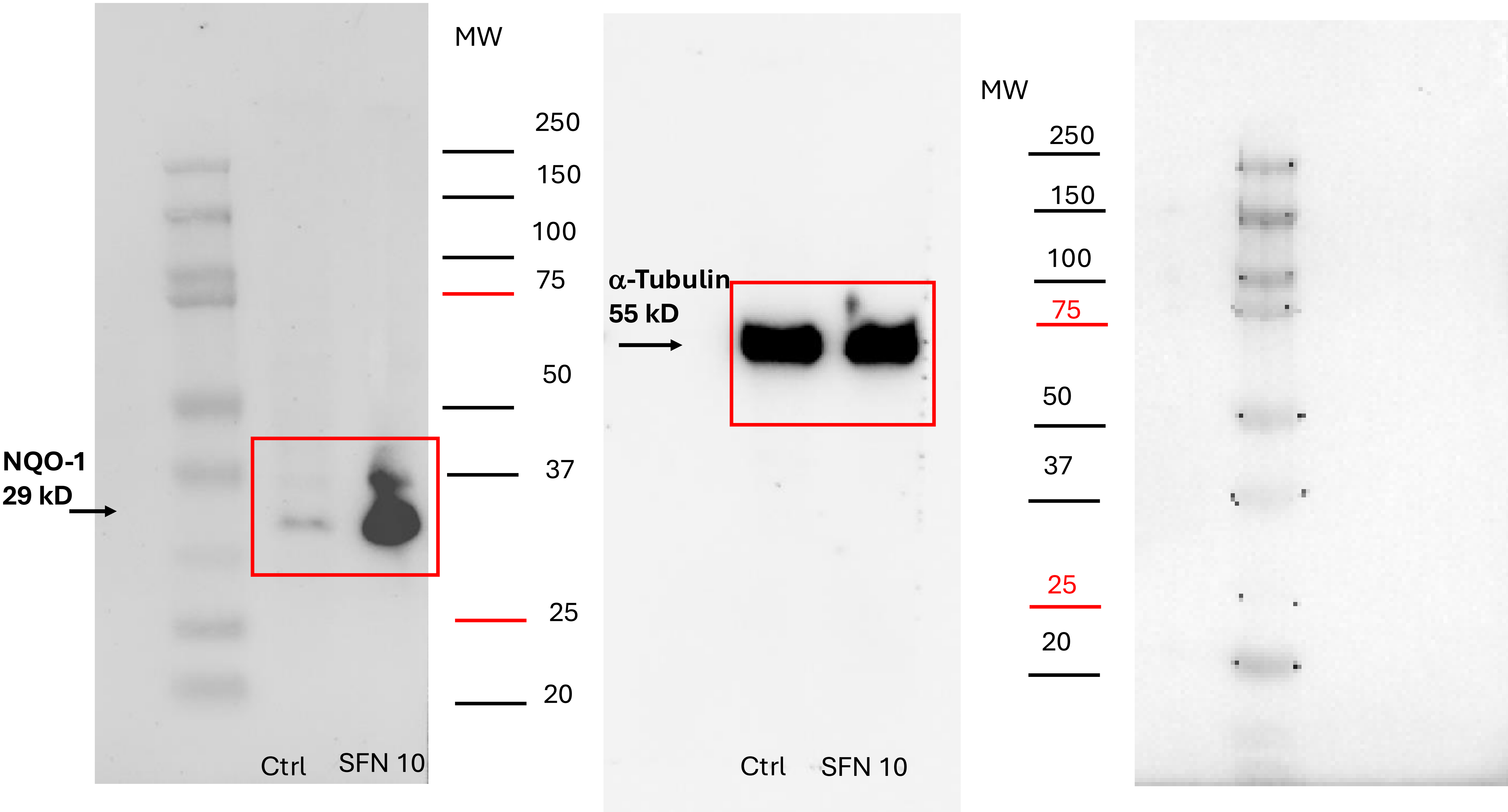

Fig. Supp 5 D Immunoblot K-562 SFN 1µM

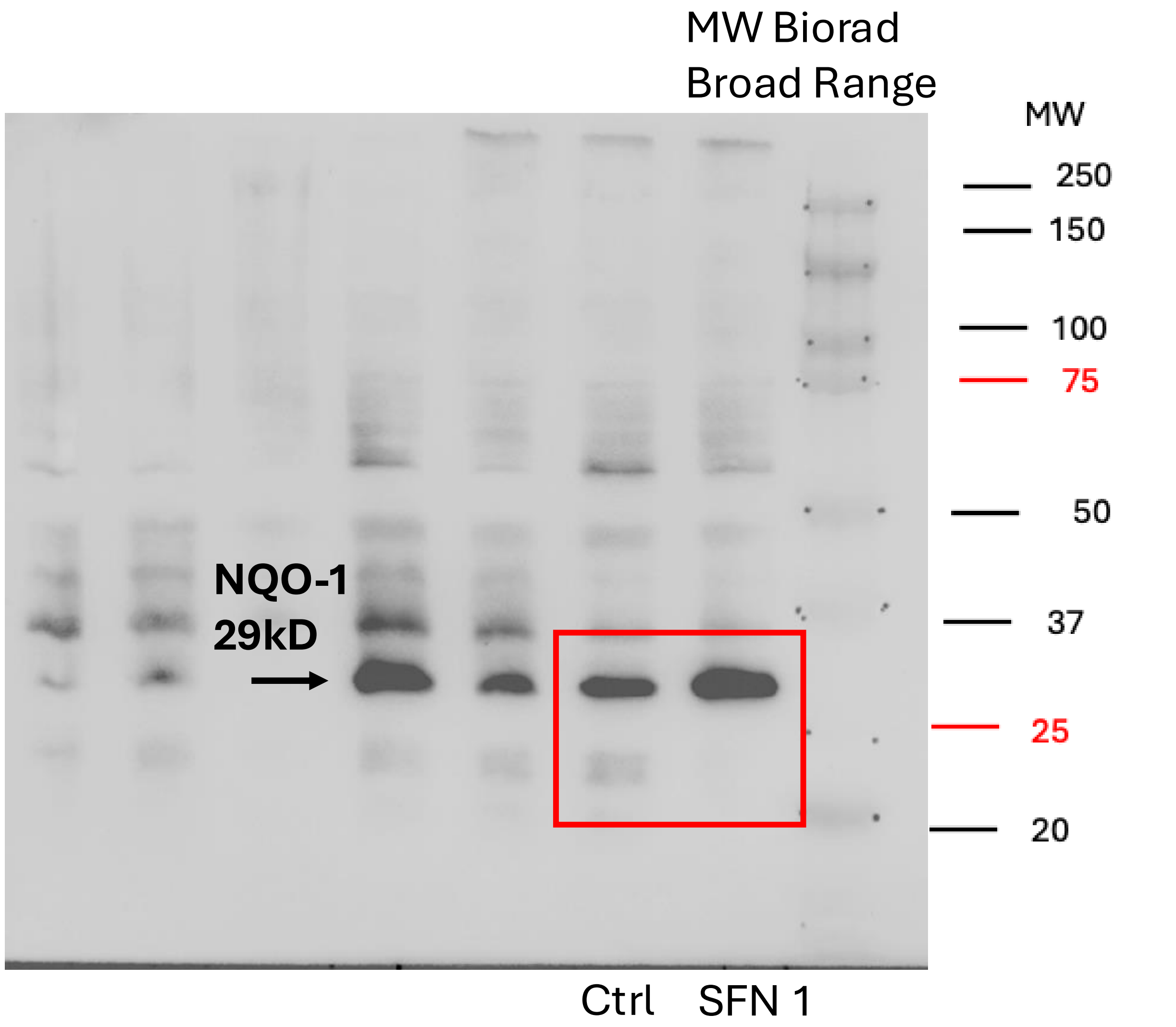

Fig. Supp 5 D Immunoblot K-562 SFN 1μM

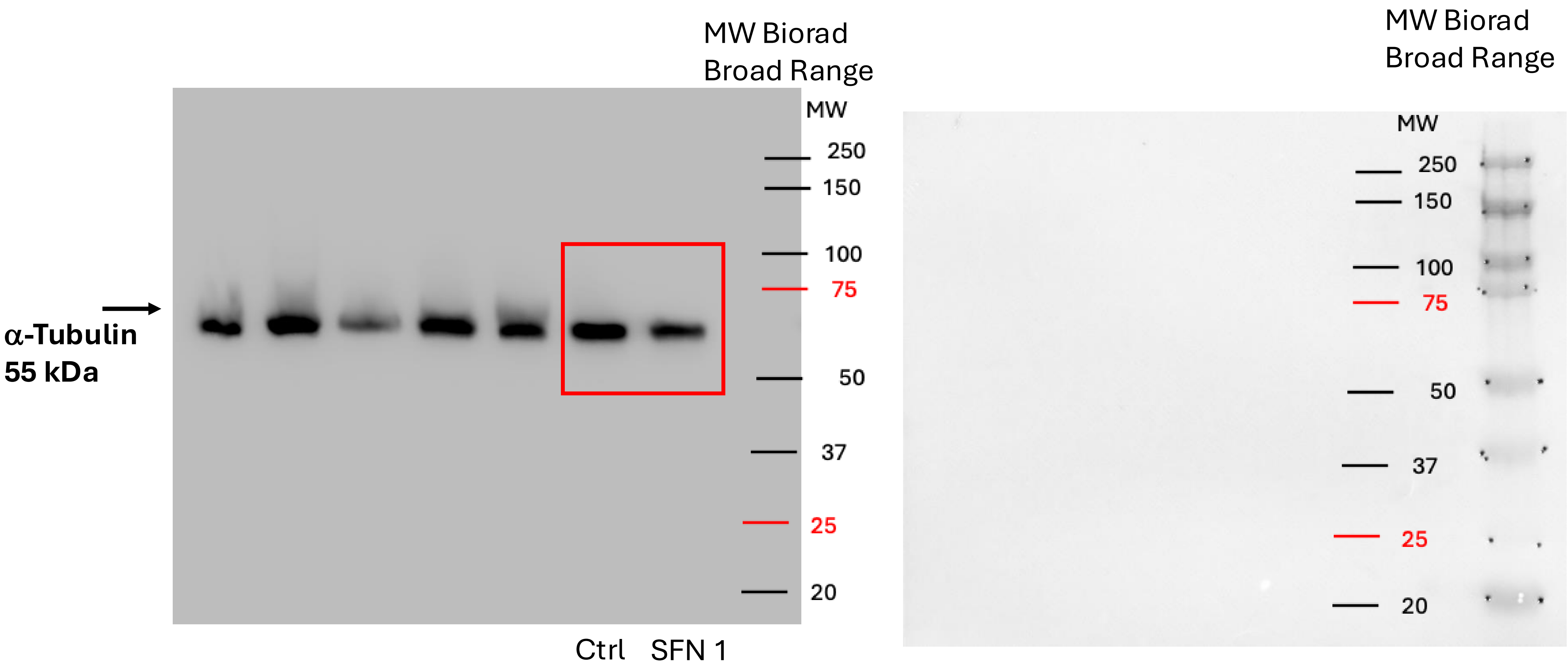

Fig. Supp 6 NQO1-Tubulin K-562 Q1

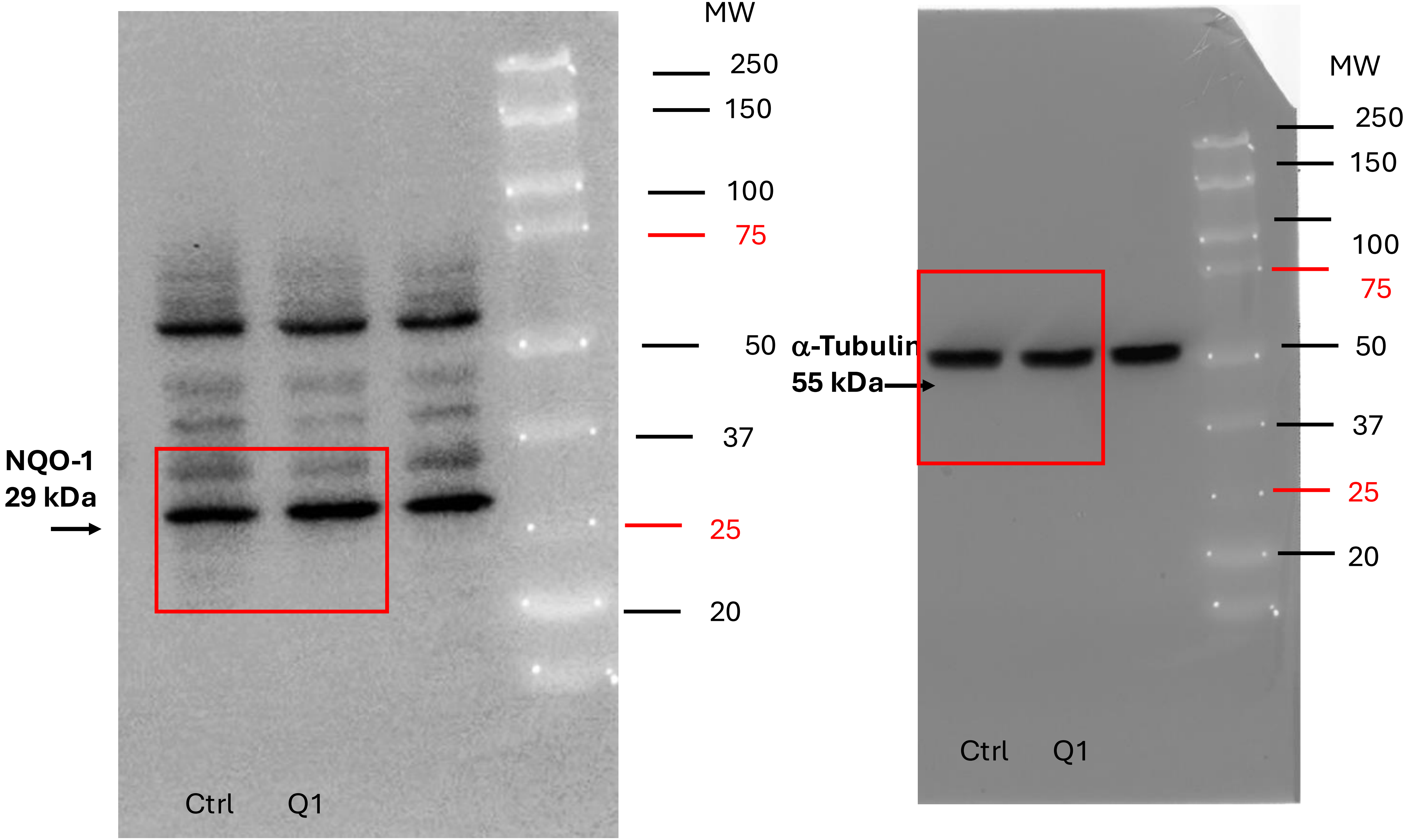

Fig. Supp 7- NQO-1-Tubulin HL-60

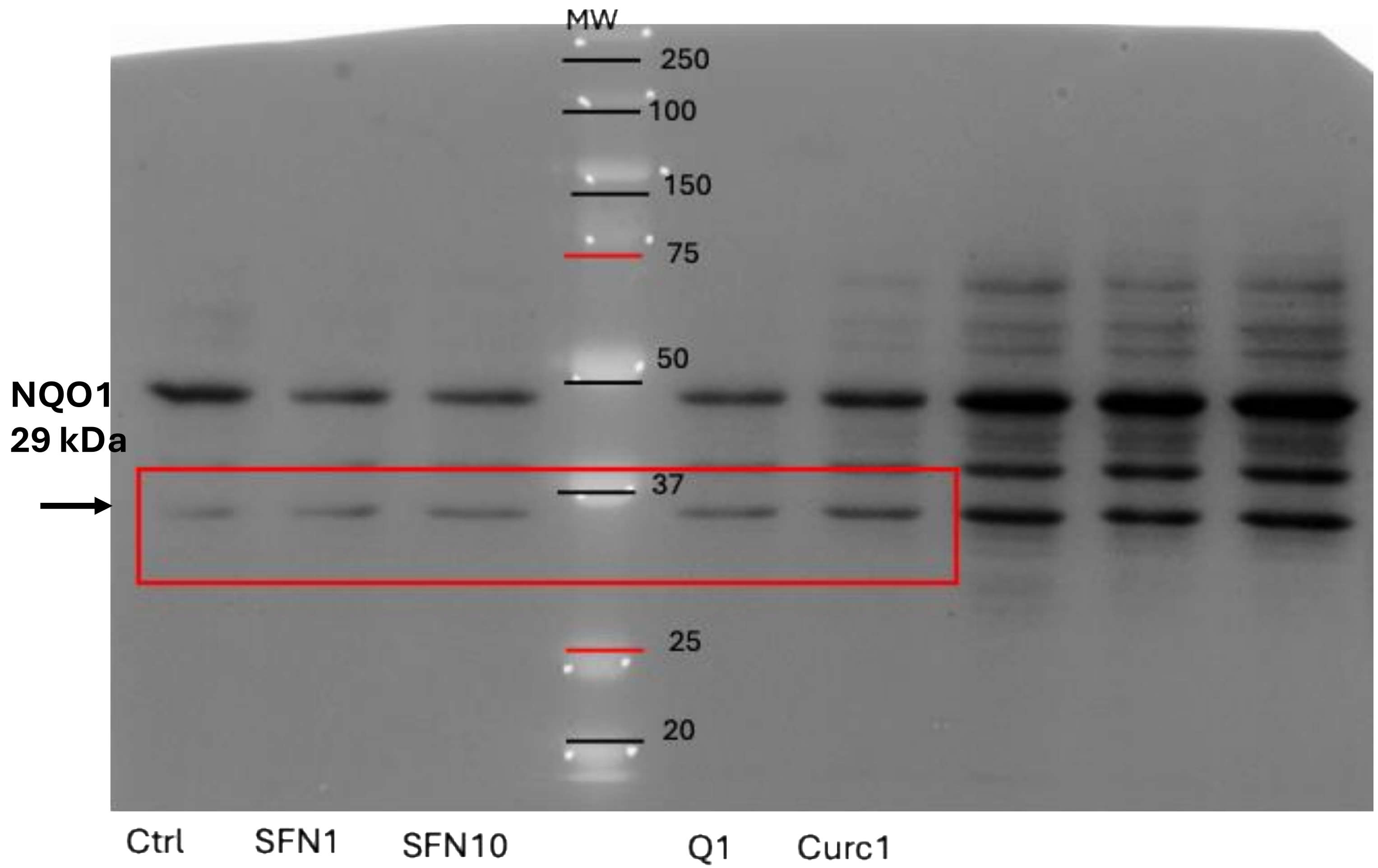

Fig. Supp 7- Tubulin HL-60

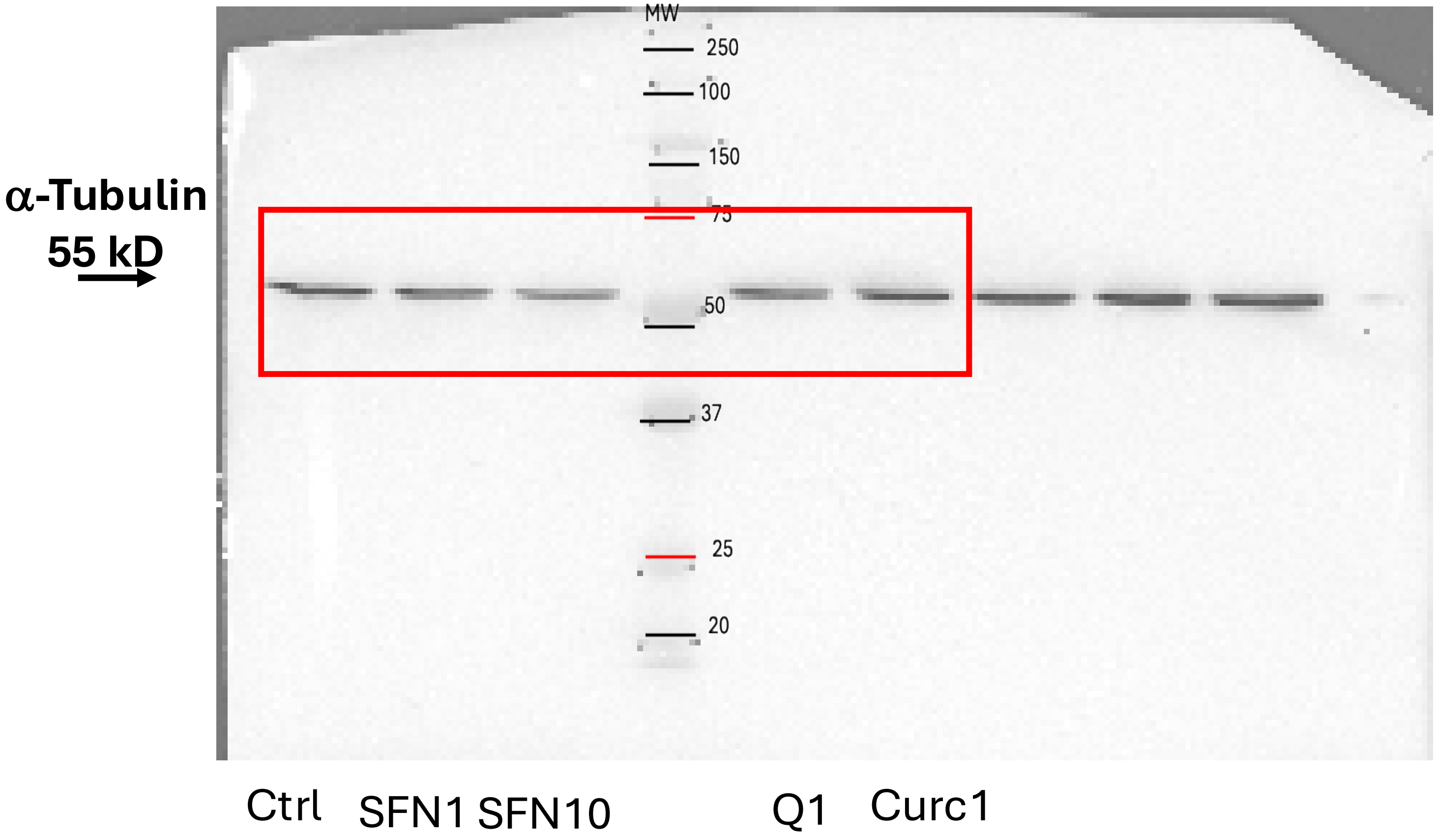

Fig. Supp 8a Tubulin-HO-1 HL-60 Curc

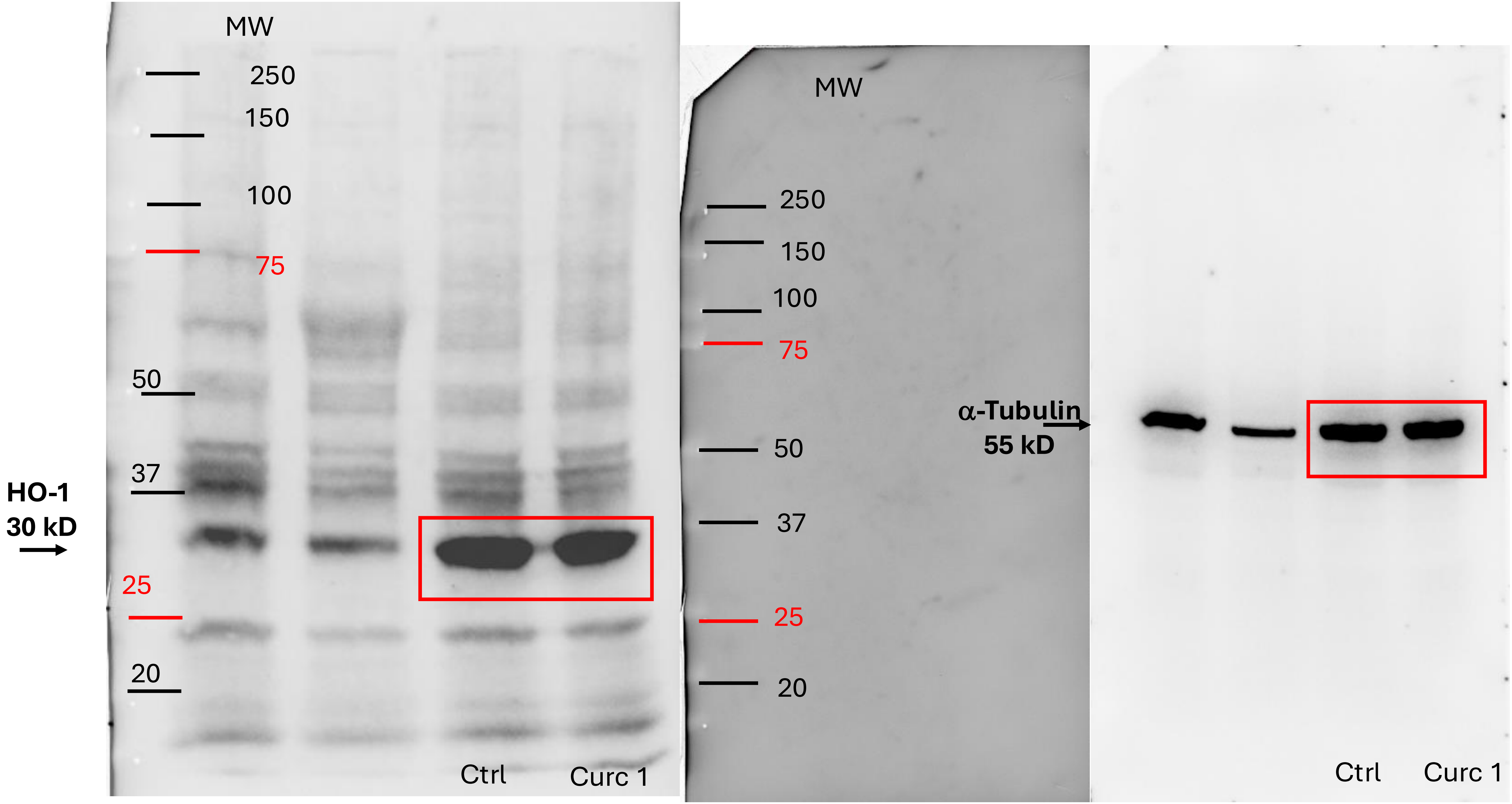

Fig. Supp 8-c HO-1-Tubulin HL-60

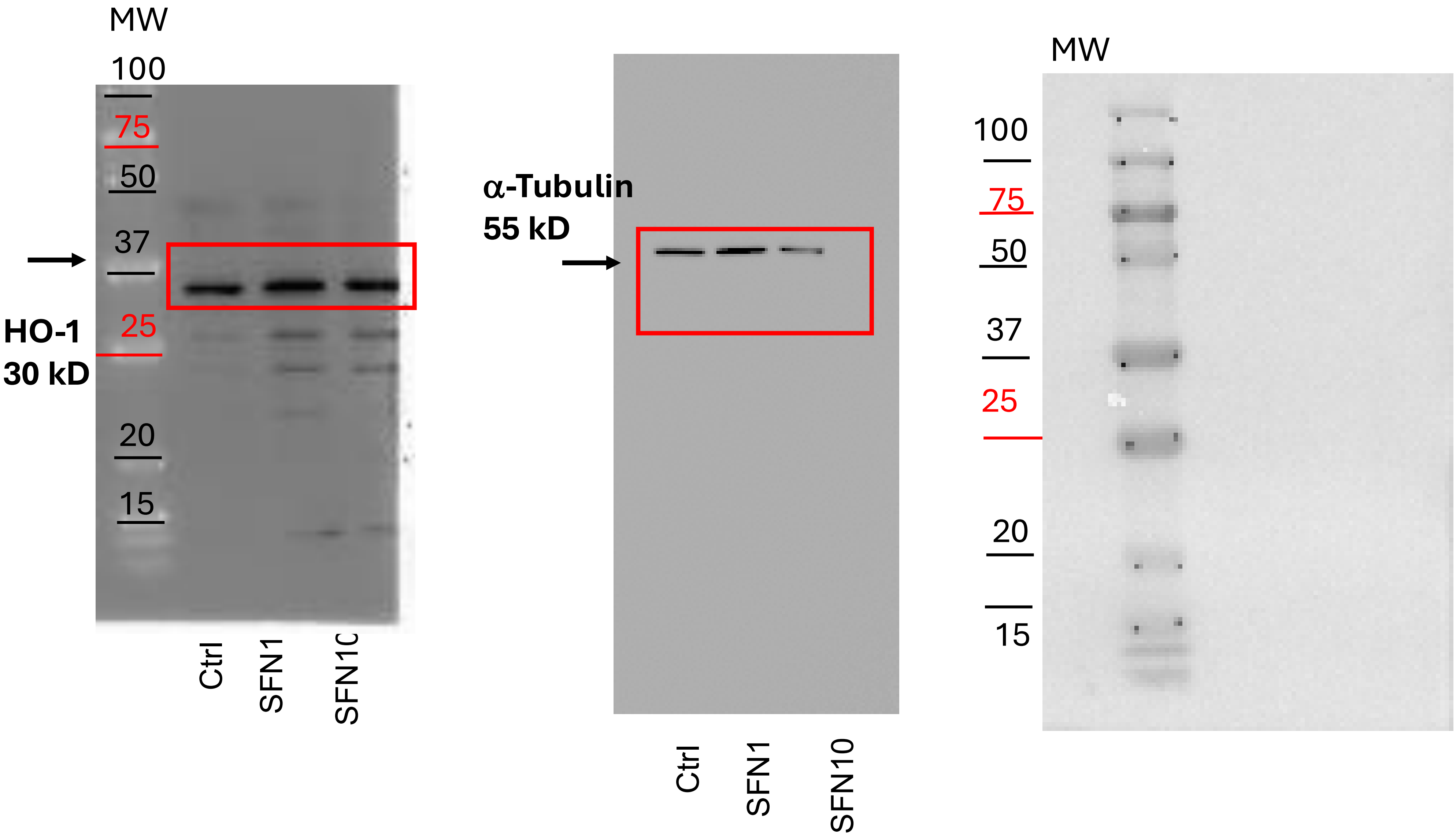

Supplement: Supplementary file 1 [file biomolecules-16-00191-s001.zip › biomolecules-4048062-supplementary.pdf]
